# Evaluating miscarriage incidence after COVID-19 vaccination

**DOI:** 10.1038/s41598-025-06904-y

**Published:** 2025-07-01

**Authors:** Fariba Shahraki-Sanavi, Sajad Sahab-Negah, Sairan Nili, Parvin Mangolian shahrbabaki, Alireza Ansari-moghaddam, Mohammad Fereidouni, Abtin Heidarzadeh, Mostafa Enayatrad, Sepideh Mahdavi, Roqayeh Aliyari, Mansooreh Fateh, Hamidreza Khajeha, Zahra Emamian, Elahe Behmanesh, Hossein Sheibani, Maryam Abbaszadeh, Reza Jafari, Maryam Valikhani, Ehsan Binesh, Hamid Vahedi, Sahar Shabestari, Reza Chaman, Hamid Sharifi, Mohammad Hassan Emamian

**Affiliations:** 1 Infectious Diseases and Tropical Medicine Research Center, Zahedan University of Medical Sciences, Zahedan, Iran; 2 Multiple Sclerosis Research Center, Neuroscience Institute, Tehran University of Medical Sciences, Tehran, Iran; 3 Social Determinants of Health Research Center, Research Institute for Health Development, Kurdistan University of Medical Sciences, Sanandaj, Iran; 4 Nursing Research Center, Razi Faculty of Nursing and Midwifery, Department of Critical Care, Kerman University of Medical Sciences, Kerman, Iran; 5 Health Promotion Research Center, Zahedan University of Medical Sciences, Zahedan, Iran; 6 Cellular and Molecular Research Center, Birjand University of Medical Sciences, Birjand, Iran; 7 School of Medicine, Guilan University of Medical Sciences, Rasht, Iran; 8 Clinical Research Development Unit, Bahar Hospital, Shahroud University of Medical Science, Shahroud, Iran; 9 Department of Epidemiology, School of Public Health, Shahroud University of Medical Sciences, Shahroud, Iran; 10 Center for Health Related Social and Behavioral Sciences Research, Shahroud University of Medical Sciences, Shahroud, Iran; 11 Ophthalmic Epidemiology Research Center, Shahroud University of Medical Sciences, Shahroud, Iran; 12 Health Technology Incubator Center, Shahroud University of Medical Sciences, Shahroud, Iran; 13 Clinical Research Development Unit, Imam Hossein Hospital, Shahroud University of Medical Science, Shahroud, Iran; 14 School of Allied Medical Sciences, Shahroud University of Medical Sciences, Shahroud, Iran; 15 HIV/STI Surveillance Research Center, WHO Collaborating Center for HIV Surveillance, Institute for Futures Studies in Health, Kerman University of Medical Sciences, Kerman, Iran

**Keywords:** Vaccine, Covid-19, Abortion, Miscarriage, Iran, Epidemiology, Infectious diseases, Reproductive disorders

## Abstract

COVID-19 infection during pregnancy might be associated with maternal complications. This study aimed to investigate the impact of COVID-19 vaccination on the risk of miscarriage. This cohort study included 26,701 women of reproductive age (15–49) who were vaccinated with different vaccines (Sinopharm, Sputnik V, AZD1222 and CoVIran Barekat) between April 2021 and August 2022 in seven cities in Iran. Among them 459 women were pregnant and included in this analysis. All pregnant women were followed up until the end of their pregnancy. The mean age (standard deviation) of pregnant women was 31.7 (6.8) years. Among them, 50 miscarriage cases occurred. The cumulative incidence of miscarriage was 10.9%; 95% confidence intervals [CI] 8.0–13.8) in total, and 11.0% (5.9–16.1), 9.7% (4.4–14.9), 12.0% (5.9–18.2), and 11.1% (4.2–18.0) for AZD1222, Sputnik V, Sinopharm and Barekat vaccines respectively. Cumulative incidence rates by vaccine brands were not statistically significant (*P* value = 0.962). The mean (SD) time interval between conception and vaccination was 3.5 (3.8) weeks and it was 7.5 (2.7) weeks for the age of the fetus at miscarriage. In general, the miscarriage rate in women of reproductive age was 4.8 (95% CI 4.1–5.7) per 1000 women. The estimated incidence rates were not higher than expected; therefore, it can be argued that COVID − 19 vaccination with Sputnik V, Sinopharm, Barekat and AZD1222 does not increase the probability of miscarriage and the vaccines are therefore safe in this respect.

## Background

Since the start of the COVID-19 pandemic, pregnant women and the potential risk of vertical transmission have become a major concern^1^. Pregnant women and their fetuses are one of the high-risk populations during the outbreak of infectious diseases^2^. Pregnant women are at risk of severe coronavirus disease 2019 (COVID-19) and acute respiratory syndrome infection by SARS-CoV-2 during pregnancy may be also be associated with an increased risk of premature birth and other adverse maternal and neonatal outcomes^1,3,4^. In a review study higher rates of intensive care unit admission, gestational diabetes, preeclampsia, Cesarean sections, and pre-term birth, were reported in women with SARS-CoV-2 infection compared to other pregnant women^5^.

Miscarriage (pregnancy loss within the first 20 weeks of conception) is a common consequence of pregnancy. Globally, there are 39 miscarriages per 1000 women aged 15–49 years^6^. Life time prevalence of at least one miscarriage was 25.7% in west Iran^7^. Obesity, smoking and alcohol consumption are among the modifiable risk factors of miscarriage, while other factors, such as uterine abnormalities, maternal infectious diseases, maternal age, and history of miscarriage, were associated with miscarriage^7–9^. The results of a systematic review showed that the risk of miscarriage increases in mothers with positive SARS-CoV-2 test results^10^. However a new systematic review and meta-analysis showed no association between SARS-CoV-2 infection and miscarriage^11^.

Following the COVID-19 epidemic, various prevention measures were implemented; among them, vaccination has a unique role. Mass vaccination against SARS-CoV-2 will eventually end the pandemic^12^. Available guidelines indicate that COVID-19 vaccination is not prohibited for pregnant women^13–15^. The American Society for Reproductive Medicine (ASRM) emphasized that “everyone, including pregnant women and those trying to become pregnant, should receive the COVID-19 vaccine^16^. Several studies have shown that vaccine hesitancy is higher among younger populations and women, which may be due to misinformation about vaccination^17,18^. In this regard, there is limited data on the estimated risk of miscarriage after COVID-19 vaccination before conception (30 days before the first day of the last menstrual cycle up to 14 days later) or during pregnancy. Few studies investigated the outcomes of pregnancy in people vaccinated for COVID-19 prevention^19–22^. Most of these studies were limited to women vaccinated with BNT162b2 and mRNA-1273 vaccines and involved a case-control design or self-reporting in an observational study. Therefore, in a cohort event monitoring study, this study investigated miscarriage in women vaccinated with AZD1222, Sputnik V, Sinopharm and CoVIran Barekat vaccines and compared the miscarriage rates by vaccine brand.

## Methods

This study was a cohort event monitoring study for safety signal detection of COVID-19 vaccines. It was based on a template, provided by the World Health Organization^23^ and its protocol published previously^24^. Briefly, a total of 93,849 individuals aged 15 years or older participated in this study, receiving one of the following vaccines: AZD1222, Sinopharm, Sputnik V, or CoVIran Barekat. The study took place in seven cities across Iran between April 2021 and August 2022. Participation in the study was voluntary and written informed consent to participate in the study was obtained from all the participants. There were no strict inclusion or exclusion criteria except for willingness to participate in the study and receipt of one of the aforementioned vaccines at the study sites. Women were enrolled regardless of pregnancy intent and could be pregnant at the time of enrolment. Contact and demographic information, history of underlying diseases and pregnancy (if any), and vaccine data including vaccine name, batch number, and time of vaccination were collected in the vaccination centers and after participants received the first dose of vaccines. Participants were followed actively by phone contacts every week. A small portion of weekly follow-ups was collected through self-reporting via a web application. Any hospitalization, COVID-19 disease, serious adverse events, and adverse events of special interest were the main outcomes followed in weekly follow ups. Participants could enter weekly follow-up information directly into the web application. If they did not submit data by the first day of the new week, it was collected actively via phone calls. Female participants were also asked about the occurrence of pregnancy. The duration of follow-up was 13 weeks after each dose of vaccines. The total follow-up period was 17 weeks for the Sinopharm, Barekat, and Sputnik vaccines, while it was 25 weeks for the AZD1222 vaccine due to the longer interval between the first and second doses of this vaccine. All hospitalized cases and participants who reported serious adverse events or adverse events of special interest were investigated by scientific committees composed of at least six medical experts to determine the role of vaccination in these events. If the women reported pregnancy during the follow-up period, follow-up continued until delivery or completion of the pregnancy, and the outcome of the pregnancy was also recorded^23,24^. The hospital records of pregnant women were also examined by scientific committees. Miscarriage was defined according to the guideline provided by the European Society of Human Reproduction and Embryology (ESHRE) as the spontaneous demise of a pregnancy until 24 weeks of gestation^25^. The age, body mass index, years of education, brand of COVID-19 vaccines, history of prior COVID-19 infection, and underlying diseases were compared between women with and without a miscarriage using t-tests, Fisher’s exact tests, and chi-square tests. Although the outcomes of pregnancy were not among the main objectives of this study, we collected more data from the incident cases of miscarriage and described them by vaccine brands.

### Sample size and statistical analysis

Assuming an 11% miscarriage rate, a type I error (α) of 0.05, and a precision (margin of error) of 0.03, the study required 418 pregnant women. The proportion of miscarriage was calculated in total and by vaccine brands with 95% confidence intervals. The comparison of demographic variables, vaccination brands, prior COVID-19 infection, and comorbidities between women with and without miscarriage done by t-test; chi-square; and Fisher exact tests. The rate of miscarriage also was calculated follow-up duration in by person-time (per 1000 women).

## Results

In this study, there were 26,701 women in the reproductive age of 15–49 years, including 459 who reported pregnancy; among them, 50 women reported miscarriage. Among the pregnant women, 31 were already pregnant at the time of enrollment. The mean (standard deviation: SD) age of the pregnant women and those with miscarriage was 31.7 (6.8), and 33.5 (6.5) years, respectively. Table 1 summarizes the characteristics of the women with miscarriage. The majority (95.5%) of miscarriages were early pregnancy losses, occurring before 12 weeks of gestation. Among the 21 women with a history of delivery, 11 (52.4%) had a previous cesarean section.

**Table 1:** The characteristics of women who had miscarriage after COVID-19 vaccination.

Descriptive variables		N*	Mean (SD)	*n* (%)
Age (year)		50	33.5 (6.5)	
Age at Menarche (year)		43	13.6 (2.0)	
Age at Marriage (year)		36	24.4 (6.2)	
Conception to vaccination interval (week)		44	4.2 (4.6)	
Gestational age (week)		44	7.6 (2.8)	
Number of lived children		44	1.0 (1.1)	
Unwanted pregnancy		44		21 (47.7)
History of infertility		44		6 (13.6)
Taking oral contraceptives		44		2 (4.5)
Using hormone medications		44		6 (13.6)
Pregnancy before vaccination		44		12 (27.3)
Admitted in hospitals		44		22 (50.0)
Number of delivery (Parity)	All women	44	1.0 (1.1)	
	0	44		19 (43.2)
	1	44		11 (25.0)
	≥ 2	44		14 (31.8)
Number of pregnancy (Gravid)	All women	44	2.4 (1.5)	
	1	44		16 (36.4)
	2	44		12 (27.3)
	≥ 3	44		16 (36.4)
Number of previous miscarriages	0	44		30 (68.2)
	1	44		10 (22.7)
	2	44		4 (9.1)

*Data for 6 miscarriage cases were unavailable

Table 2 compares the demographic, vaccination status and comorbidities between the women whose pregnancies ended with miscarriage and those with live births. Among the studied variables there were no statistically significant differences between the two groups.

**Table 2:** Comparison of demographic, COVID-19 vaccination, and comorbidities among pregnant women by miscarriage status.

Independent variables	Miscarriage		*P* value
	Yes (*n* = 50)	No (*n* = 409)	
Age (Year)	33.5 (6.5)	31.5 (6.8)	0.056^†^
Body mass index (Kg/m^2^)	24.7 (3.9)	25.1 (4.3)	0.562^†^
Education (year)	14.2 (2.8)	13.6 (3.7)	0.264^†^
COVID-19 vaccination			
Sputnik V	12 (24.0)	112 (27.4)	0.953^‡^
CoVIran Barekat	9 (18.0)	72 (17.6)	
AZD1222	16 (32.0)	130 (31.8)	
Sinopharm	13 (26.0)	95 (23.2)	
Prior COVID-19 disease	12 (24.0)	120 (29.3)	0.431^‡^
Underlying diseases			
Hypertension	2 (4.0)	4 (1.0)	0.136^§^
Hypothyroidism	2 (4.0)	22 (5.4)	0.502^§^
Allergy	0	11 (2.7)	1.0^§^
Cancer	0	1 (0.2)	1.0^§^
Respiratory	0	4 (1.0)	1.0^§^
Cardiac	0	3 (0.7)	1.0^§^
Hepatic	0	2 (0.5)	1.0^§^
Renal	0	1 (0.2)	1.0^§^
Diabetes	0	4 (1.0)	1.0^§^

†, t-test; ‡, chi-square; §, Fisher exact test

**Fig. 1:**
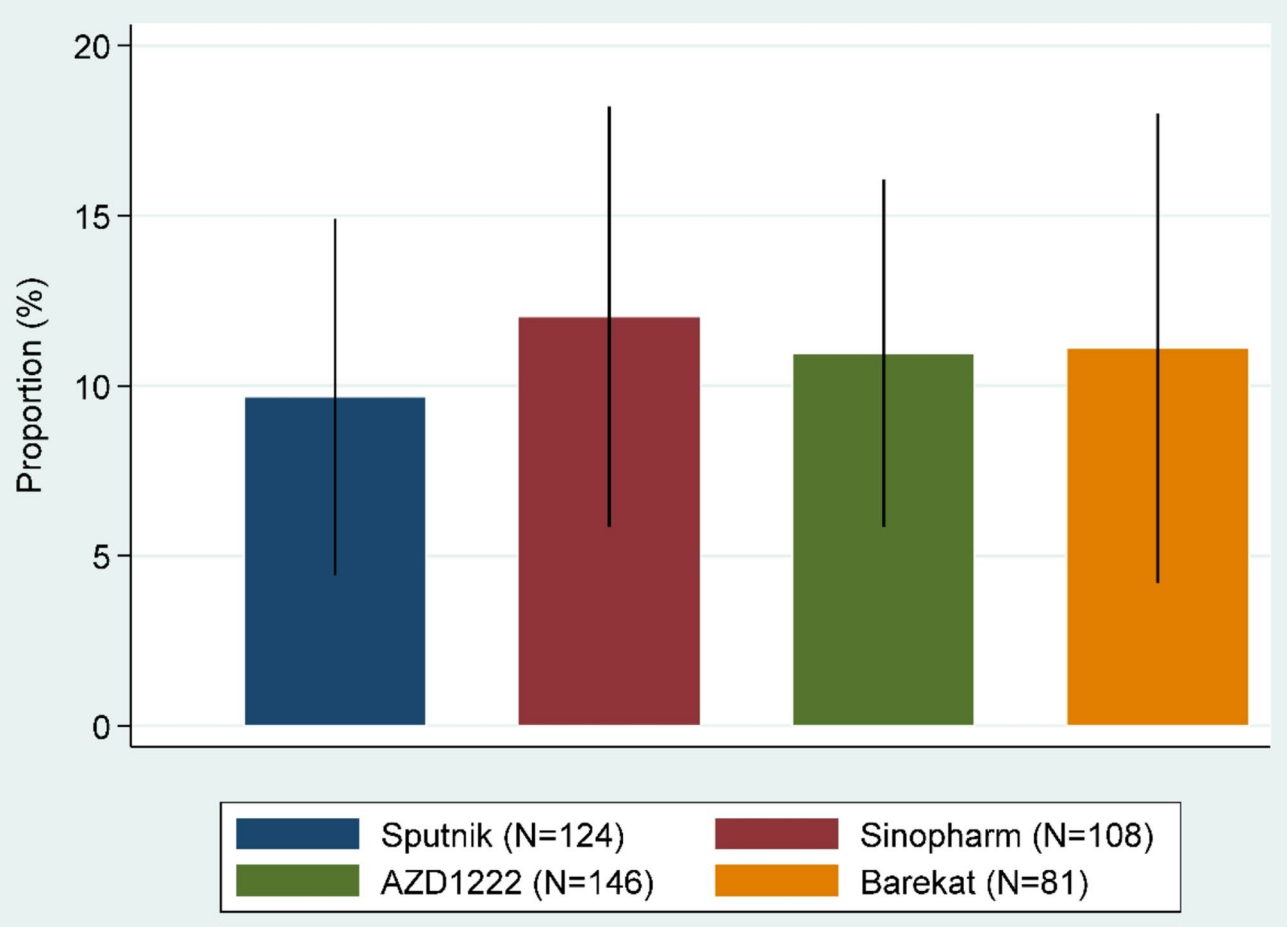
The proportion of miscarriage in vaccinated women by vaccine brands. Error bars indicate 95% confidence intervals.

The proportion of miscarriage in this study was 10.9% (95% CI 8.0–13.8). The miscarriage proportions were 9.7%, 12.0%, 11.0%, and 11.1% in women who were vaccinated with Sputnik V, Sinopharm, AZD1222, and Barekat respectively. The difference in miscarriage proportions by vaccine brands was not statistically significant (Fig. 1). Power analysis indicated that with a proportion of 11% and a sample size of 459, there would be 89.4% power to detect a 0.05 difference (delta) between the alternative and null values. When the effect size (Δ) was reduced to 0.04, the statistical power decreased to 75.3%. Therefore, the sample size of 459 provided adequate statistical power (75–89%) to detect the true miscarriage proportion across the specified effect sizes.

The rate of miscarriage was 4.8 (95% CI 4.1–5.7) per 1000 women aged 15–49 years. This rate was slightly higher for women who were vaccinated with Barekat, although the differences were not statistically significant (Table 3).

**Table 3:** The annual rates of miscarriage per 1000 women aged 15–49 years old who were vaccinated with COVID-19 vaccines.

Vaccine brands	*n*	Mean (SD) of age in year	Number and (Incidence rate/1000) of pregnancies	Observed miscarriage cases	Estimated miscarriage cases in 1 year	Annual miscarriage rate per 1000 (95% CI)
Sputnik V	8226	32.1 (7.8)	124 (15.1)	12	37	4.5 (3.2–6.2)
Sinopharm	8728	32.7 (11.4)	108 (12.4)	13	40	4.6 (3.4–6.2)
AZD1222	6986	33.2 (7.9)	146 (20.1)	16	33	4.7 (3.3–6.6)
CoVIran Barekat	4756	36.1 (8.5)	81 (17.0)	9	28	5.9 (3.9–8.5)
Total	28,696	33.2 (9.3)	459 (16.0)	50	138	4.8 (4.1–5.7)

CI, confidence intervals

## Discussion

In this cohort event monitoring study with active weekly follow-ups, 50 out of 459 (10.9%) women who were vaccinated with COVID-19 vaccines reported miscarriage. This proportion of miscarriage is lower than the most recent estimates for Iran (16.8%) and the North Africa and Middle East region (19.8%) in 2019^26^. The reported rate of miscarriage per 1000 women aged 15–49 years old (4.8) is also lower than the old estimates from many countries such as the US (20.8), UK (17.0), Canada (15.2), Japan (12.3) and Bahrain (11.0)^27^ and also from a recent global estimate (39.0)^6^. The estimated rate of miscarriage is almost similar to other studies in Iran^28–30^; therefore, it can be concluded that the COVID-19 vaccines, investigated in this study were not associated with the risk of miscarriage in pregnant women. Abortion rates in Iran may be underestimated due to the illegality of induced abortion and the low visibility of such procedures^31^. However, the reporting of miscarriage (spontaneous abortion) is likely unaffected by these circumstances. The miscarriage rate in this study is much lower than in other countries and even from predicted rates by statistical models in Iran^32^. On the other hand, we actively follow the outcomes of pregnancy, which may lead to a more accurate estimate for the miscarriage rate. The other factor that may influence the low miscarriage rate in this study may be concerns of families about COVID-19 vaccines which led to a lower rate of pregnancy and a higher rate (47.7%) of unwanted pregnancies.

Consistent with the present study, a population-based, case-control study in the United States that investigated miscarriage after COVID-19 vaccination in early pregnancy^21^, did not find evidence of an increased probability of miscarriage due to COVID-19 vaccination. The result of another observational retrospective study showed that the odds of spontaneous abortion were non-significant for both versions of the mRNA COVID-19 vaccine (Pfizer and Moderna)^20^. In a study using public data in Thailand, the incidence of abortion was 2.19 per 100,000 doses of mRNA vaccines administered^33^. Based on the results of a descriptive study at New York University on 424 pregnant women who had received one dose of the mRNA COVID-19 vaccine, the proportion of spontaneous abortion was 6.5% during the first trimester after vaccination^34^. One study using data from the CDC showed a 14.1% (12.1–16.1) risk of spontaneous abortion at 6–20 weeks of pregnancy in women who had received a single dose of the mRNA COVID-19 vaccine^35^. In addition, the results of a case-control study on first-trimester pregnancies after COVID-19 vaccination in Norway did not find any evidence of an increased risk of early pregnancy loss after COVID-19 vaccination^36^. A lower risk of miscarriage has also been reported following a single dose of COVID-19 vaccination during pregnancy^37^. Additionally, systematic reviews and meta-analyses have found no association between COVID-19 vaccination during pregnancy and miscarriage^38–40^. Overall, all studies indicated that the risk of spontaneous abortion after immunization during pregnancy was proportionate with the risk in non-vaccinated pregnant women^41^.

Other researches show that most spontaneous abortions occur in the first trimester of pregnancy (first 12 weeks)^19,42^. Similarly, in the current study, most miscarriages occurred up to the twelfth week. Moreover, 30% of pregnancies are lost between implantation and the sixth week of pregnancy^43^. Consistently, 12 out of 50 women in our study reported a miscarriage before the sixth week. In addition, the risk of spontaneous abortion increases with age, being 10–20% in pregnant women under 25 years and rising to 60–70% in those over 40 years^44^. Therefore, compared to AZD1222 and Sputnik, slightly higher rates of miscarriage in women who were vaccinated with Sinopharm and Barekat, can be attributed to the higher age of these groups. It has also been reported that the rate of miscarriage increases after a previous spontaneous abortion^45^. In women with a history of recurrent abortions, the probability of abortion is 24%, 30%, and 40–50% after two, three, and four abortions, respectively^46^. In the current study, about one-third of the women had a history of miscarriage, which explains the miscarriages recorded in these participants and makes the role of COVID-19 vaccination in miscarriages less prominent.

The COVID-19 pandemic significantly impacted obstetric healthcare utilization, as evidenced by global studies. Pregnant women diagnosed with COVID-19 had higher rates of adverse outcomes^47^. In Italy, emergency obstetric admissions declined overall, though critical cases rose^48^, while Greece saw reduced surgical emergency visits during lockdowns without changes in total hospitalizations^49^. Notably, vaccinated pregnant women in Iran had lower COVID-19-related hospitalization rates than their unvaccinated counterparts^50^, yet adverse outcomes like preterm birth and stillbirth increased during the pandemic^51^. These trends suggest that pandemic-related disruptions altered healthcare-seeking behaviors, particularly among pregnant women, who may have delayed non-urgent care due to fear of infection or restrictions. However, conditions requiring immediate intervention, such as miscarriage, likely remained stable in hospitalization rates due to their urgent nature. Together, these findings underscore the pandemic’s heterogeneous effects on maternal health services, emphasizing the need for careful follow-up of participants in related researches.

Active weekly follow-ups in a cohort event monitoring study, comparing miscarriage rates in recipients of four different COVID-19 vaccines, for which there is insufficient evidence, and sufficient sample size, were the strengths of the current study. However, six of the 50 women who had a miscarriage chose not to provide additional information, such as the number of pregnancies, number of deliveries, and history of previous miscarriages. This may be considered a limitation of the study, although it does not influence the reported miscarriage rate, which is the main objective of the current research. Another limitation is that some women may not have disclosed their pregnancies or miscarriages, as pregnancy status was self-reported and not universally confirmed by testing. Finally, it is clear that not all risk factors for miscarriage have been investigated in this study. However, given that COVID-19 vaccination encompasses all population groups, selection bias is not a concern in the current research.

## Conclusion

According to the results of the present study, 10.9% of pregnancies in women who received COVID-19 vaccines resulted in miscarriage, which is not more than global and local estimates, and it can be concluded that vaccination against COVID-19 with Sputnik V, Sinopharm, Barekat and AZD1222 vaccines does not increase the risk of miscarriage and these vaccines are safe in this regard.

## Data Availability

The datasets generated and/or analyzed during the current study are not publicly available due to ongoing reports that are not yet prepared, but are available from the corresponding author on reasonable request.

## Abbreviations

COVID-19: Coronavirus Disease 2019
SARS-CoV-2: Severe Acute Respiratory Syndrome Coronavirus 2
SD: Standard Deviation
CI: Confidence Intervals
CDC: Centers for Disease Control and Prevention

## References

[1] Qiao, J. What are the risks of COVID-19 infection in pregnant women? *Lancet* **395** (10226), 760–762 (2020). 32151334 10.1016/S0140-6736(20)30365-2 PMC7158939

[2] Alfaraj, S. H., Al-Tawfiq, J. A. & Memish, Z. A. Middle East respiratory syndrome coronavirus (MERS-CoV) infection during pregnancy: Report of two cases & review of the literature. *J. Microbiol. Immunol. Infect.* **52** (3), 501–503 (2019). 29907538 10.1016/j.jmii.2018.04.005 PMC7128238

[3] Allotey, J. et al. Clinical manifestations, risk factors, and maternal and perinatal outcomes of coronavirus disease 2019 in pregnancy: Living systematic review and meta-analysis. *BMJ* **370**, m3320 (2020). 32873575 10.1136/bmj.m3320 PMC7459193

[4] Bobei, T. I. et al. The impact of SARS-CoV-2 infection on premature Birth—Our experience as COVID center. *Medicina* **58** (5), 587 (2022). 35630005 10.3390/medicina58050587 PMC9146843

[5] Oltean, I. et al. Impact of SARS-CoV-2 on the clinical outcomes and placental pathology of pregnant women and their infants: A systematic review. *Heliyon* **7** (3), e06393 (2021). 33688585 10.1016/j.heliyon.2021.e06393 PMC7923950

[6] Bearak, J. et al. Unintended pregnancy and abortion by income, region, and the legal status of abortion: Estimates from a comprehensive model for 1990–2019. *Lancet Glob Health*. **8** (9), e1152–e1161 (2020). 32710833 10.1016/S2214-109X(20)30315-6

[7] Moradinazar, M. et al. Lifetime prevalence of abortion and risk factors in women: Evidence from a cohort study. *J. Pregnancy*. **2020**, 4871494 (2020). 32395342 10.1155/2020/4871494 PMC7201453

[8] Sundermann, A. C. et al. Alcohol use in pregnancy and miscarriage: A systematic review and meta-analysis. *Alcohol Clin. Exp. Res.* **43** (8), 1606–1616 (2019). 31194258 10.1111/acer.14124 PMC6677630

[9] Pineles, B. L., Park, E. & Samet, J. M. Systematic review and metaanalysis of miscarriage and maternal exposure to tobacco smoke during pregnancy. *Am. J. Epidemiol.* **179** (7), 807–823 (2014). 24518810 10.1093/aje/kwt334 PMC3969532

[10] Kazemi, S. N. et al. COVID-19 and cause of pregnancy loss during the pandemic: A systematic review. *PLoS ONE*. **16** (8), 0255994 (2021). 10.1371/journal.pone.0255994 PMC8357105 34379700

[11] van Baar, J. A. C. et al. COVID-19 in pregnant women: A systematic review and meta-analysis on the risk and prevalence of pregnancy loss. *Hum. Reprod. Update*. **30** (2), 133–152 (2024). 38016805 10.1093/humupd/dmad030 PMC10905512

[12] Viana, J. et al. Controlling the pandemic during the SARS-CoV-2 vaccination rollout. *Nat. Commun.* **12** (1), 1–5 (2021). 34135335 10.1038/s41467-021-23938-8 PMC8209021

[13] Centers for Disease Control and Prevention. COVID-19 vaccines: Interim clinical considerations for use of COVID-19 vaccines currently authorized in the United States. (2021). (https://www.cdc.gov/vaccines/covid-19/info-by-product/clinicalconsiderations.html)

[14] American College of Obstetricians and Gynaecologists. Vaccinating pregnant and lactating patients against COVID-19: Practice advisory. December (2020). https://www.acog.org/clinical/clinical-guidance/practice-advisory/articles/2020/12/vaccinating-pregnant-and-lactating-patients-against-covid-19).

[15] American Academy of Pediatrics. Interim guidance for COVID-19 vaccination in children and adolescents. (2021). https://services.aap.org/en/pages/2019-novel-coronavirus-covid-19-infections/clinical-guidance/interim-guidance-for-covid-19-vaccination-in-children-and-adolescents/).

[16] The American Society for Reproductive Medicine the American College of Obstetricians and Gynaecologists, The Society for Maternal-Fetal Medicine. Medical experts continue to assert that COVID vaccines do not impact fertility. (2021). https://www.asrm.org/news-and-publications/news-and-research/pressreleases-and-bulletins/asrm-smfm-acog-issue-joint-statementmedical-experts-continue-to-assert-that-covid-vaccines-do-notimpact-fertility/

[17] Troiano, G. & Nardi, A. Vaccine hesitancy in the era of COVID-19. *Public. Health*. **194**, 245–251 (2021). 33965796 10.1016/j.puhe.2021.02.025 PMC7931735

[18] Dror, A. A. et al. Vaccine hesitancy: The next challenge in the fight against COVID-19. *Eur. J. Epidemiol.* **35** (8), 775–779 (2020). 32785815 10.1007/s10654-020-00671-y PMC8851308

[19] Shimabukuro, T. T. et al. Preliminary findings of mRNA Covid-19 vaccine safety in pregnant persons. *New. Engl. J. Med.* **384** (24), 2273–2282 (2021). 33882218 10.1056/NEJMoa2104983 PMC8117969

[20] Citu, I. M. et al. The risk of spontaneous abortion does not increase following first trimester mRNA COVID-19 vaccination. *J. Clin. Med.* **11**(6), 1698 (2022). 35330023 10.3390/jcm11061698 PMC8955378

[21] Kharbanda, E. O. et al. Spontaneous abortion following COVID-19 vaccination during pregnancy. *JAMA* **326** (16), 1629–1631 (2021). 34495304 10.1001/jama.2021.15494 PMC8427483

[22] Favre, G. et al. COVID-19 mRNA vaccine in pregnancy: Results of the Swiss COVI-PREG registry, an observational prospective cohort study. *Lancet Reg. Health Eur.* **18**, 100410 (2022). 35651954 10.1016/j.lanepe.2022.100410 PMC9148537

[23] World Health Organization. Protocol template to be used as template for observational study protocols: Sentinel surveillance of adverse events of special interest (AESIs) after vaccination with COVID-19 vaccines 2021 [Available from: https://apps.who.int/iris/bitstream/handle/10665/342194/9789240029507-eng.pdf?sequence=1]

[24] Aliyari, R. et al. Study protocol: Cohort event monitoring for safety signal detection after vaccination with COVID-19 vaccines in Iran. *BMC Public. Health*. **22** (1), 1153 (2022). 35681132 10.1186/s12889-022-13575-1 PMC9178529

[25] European Society of Human Reproduction and Embryology (ESHRE). Guideline on the management of recurrent pregnancy loss. (2023). Available from: https://www.eshre.eu/Guidelines-and-Legal/Guidelines/Recurrent-pregnancy-loss. Accessed: 26 Oct 2024.

[26] Moradinazar, M. et al. Epidemiological features of spontaneous abortion in the North Africa and the middle East from 1990 to 2019: Results from the global burden of disease study 2019. *J. Family Reprod. Health*. **16** (3), 183–191 (2022). 36569256 10.18502/jfrh.v16i3.10579 PMC9759432

[27] World Population Review, Abortion Rates by Country. (2022). Available at: https://worldpopulationreview.com/country-rankings/abortion-rates-by-country, Accessed 11 Dec 2022.

[28] Erfani, A. Levels, trends and correlates of abortion in Tehran, Iran: 2009–2014. *Int. Perspect. Sex. Reprod. Health*. **42** (2), 93–101 (2016). 28825910 10.1363/42e1316

[29] Motaghi, Z. et al. Induced abortion rate in Iran: A meta-analysis. *Arch. Iran. Med.* **16** (10), 594–598 (2013). 24093141

[30] Erfani, A. Induced abortion in Tehran, Iran: Estimated rates and correlates. *Int. Perspect. Sex. Reprod. Health*. **37** (3), 134–142 (2011). 21988789 10.1363/3713411

[31] Zamanian, M. et al. Estimating the visibility rate of abortion: a case study of kerman, Iran. *BMJ Open.* **6**, e012761 (2016). 27737886 10.1136/bmjopen-2016-012761 PMC5073643

[32] Zamanian, M., Zolala, F., Haghdoost, A. A. & Baneshi, M. R. Estimating the annual abortion rate in kerman, Iran: comparison of direct, network Scale-Up, and single sample count methods. *Int. J. Fertil. Steril.* **13** (3), 209–214 (2019). 31310075 10.22074/ijfs.2019.5721 PMC6642432

[33] Sookaromdee, P. & Wiwanitkit, V. Magnitude of abortion after COVID-19 vaccination: how about rate? *Erciyes Med. J.* **44** (2), 244–245 (2022).

[34] Trostle, M. E. et al. COVID-19 vaccination in pregnancy: early experience from a single institution. *Am. J. Obstet. Gynecol. MFM*. **3** (6), 100464 (2021). 34411758 10.1016/j.ajogmf.2021.100464 PMC8366042

[35] Zauche, L. H. et al. Receipt of mRNA Covid-19 vaccines and risk of spontaneous abortion. *New. Engl. J. Med.* **385** (16), 1533–1535 (2021). 34496196 10.1056/NEJMc2113891 PMC8451181

[36] Magnus, M. C. et al. Covid-19 vaccination during pregnancy and first-trimester miscarriage. *New. Engl. J. Med.* **385** (21), 2008–2010 (2021). 34670062 10.1056/NEJMc2114466 PMC8552533

[37] Wang, J., Deng, Y. & Wang, W. COVID-19 vaccination during pregnancy and adverse perinatal outcomes: a systematic review and meta-analysis. *Trans. R Soc. Trop. Med. Hyg.* **118** (7), 405–425 (2024). 38291854 10.1093/trstmh/trad093

[38] Ding, C. et al. Associations of COVID-19 vaccination during pregnancy with adverse neonatal and maternal outcomes: A systematic review and meta-analysis. *Front. Public. Health*. **11**, 1044031 (2023). 36794075 10.3389/fpubh.2023.1044031 PMC9922836

[39] Rahmati, M. et al. Effects of COVID-19 vaccination during pregnancy on SARS-CoV-2 infection and maternal and neonatal outcomes: A systematic review and meta-analysis. *Rev. Med. Virol.* **33** (3), e2434 (2023). 36896895 10.1002/rmv.2434

[40] Ciapponi, A. et al. Safety and effectiveness of COVID-19 vaccines during pregnancy: A living systematic review and Meta-analysis. *Drug Saf.* **47** (10), 991–1010 (2024). 39009928 10.1007/s40264-024-01458-w PMC11399161

[41] Rimmer, M. P., Teh, J. J., Mackenzie, S. C. & Al Wattar, B. H. The risk of miscarriage following COVID-19 vaccination: A systematic review and meta-analysis. *Hum. Reprod.* **38** (5), 840–852 (2023). 36794918 10.1093/humrep/dead036 PMC10152171

[42] The Johns Hopkins University School of Medicine, Department of Gynecology. The Johns Hopkins Manual of Gynecology and Obstetrics; (LWW, 2010).

[43] Jeve, Y. B. & Davies, W. Evidence-based management of recurrent miscarriages. *J. Hum. Reprod. Sci.* **7** (3), 159–169 (2014). 25395740 10.4103/0974-1208.142475 PMC4229790

[44] Cohain, J. S., Buxbaum, R. E. & Mankuta, D. Spontaneous first trimester miscarriage rates per woman among Parous women with 1 or more pregnancies of 24 weeks or more. *BMC Pregnancy Childbirth*. **17** (1), 437 (2017). 29272996 10.1186/s12884-017-1620-1 PMC5741961

[45] Kashanian, M., Akbarian, A. R., Baradaran, H. & Shabandoust, S. H. Pregnancy outcome following a previous spontaneous abortion (miscarriage). *Gynecol. Obstet. Invest.* **61** (3), 167–170 (2006). 16428886 10.1159/000091074

[46] Azargoon, A., Heidary, S. & Alavi Toussy, J. Comparaing the causes of abortion in patients with two or more than two consecutive miscarriages. *Tehran Univ. Med. J.* **69** (4), 245–252 (2011).

[47] Villar, J. et al. Maternal and neonatal morbidity and mortality among pregnant women with and without COVID-19 infection: The INTERCOVID multinational cohort study. *JAMA Pediatr.* **175** (8), 817–826 (2021). 33885740 10.1001/jamapediatrics.2021.1050 PMC8063132

[48] Riemma, G. et al. Obstetric and gynecological admissions and hospitalizations in an Italian Tertiary-Care hospital during COVID-19 pandemic: A retrospective analysis according to restrictive measures. *J. Clin. Med.* **12** (22), 7097 (2023). 38002709 10.3390/jcm12227097 PMC10672011

[49] Pikoulis, E. et al. The effect of the COVID pandemic lockdown measures on surgical emergencies: Experience and lessons learned from a Greek tertiary hospital. *World J. Emerg. Surg.* **16** (1), 22 (2021). 33962622 10.1186/s13017-021-00364-1 PMC8102842

[50] Changizi, N. et al. Vaccination effects on reducing COVID-19 complications in pregnancy: A large-scale report from Iran. *Int. J. Gynaecol. Obstet.* **163** (3), 1012–1017 (2023). 37655467 10.1002/ijgo.15077

[51] Farhadi, R., Noori, H., GhaffariSaravi, V. & Moosazadeh, M. Stillbirth and preterm birth during lockdown periods in 5 waves of COVID-19 pandemic in Northern Iran: A Region-Wide cohort study in Mazandaran Province. *Health Serv. Res. Manag. Epidemiol.* **18**, 10:23333928231180561 (2023). 10.1177/23333928231180561 PMC10280785 37347050

